# Cognitive assessment in preterms by Bayley-III: development in the first year and associated factors

**DOI:** 10.1590/1984-0462/2024/42/2022164

**Published:** 2023-08-25

**Authors:** Suelen Rosa de Oliveira, Ana Carolina Cabral de Paula Machado, Lívia de Castro Magalhães, Débora Marques de Miranda, Jonas Jardim de Paula, Maria Cândida Ferrrez Bouzada

**Affiliations:** a Universidade Federal de Minas Gerais, Belo Horizonte, Minas Gerais, Brasil.

**Keywords:** Infant premature, Child development, Cognition, Cognition disorders, Prematuro, Desenvolvimento infantil, Cognição, Transtornos cognitivos

## Abstract

**Objective:**

To analyze the cognitive development of preterm infants at six and 12 months of corrected age and the associations with perinatal and socioeconomic factors.

**Methods:**

Cognitive development of 40 infants (20 preterm and 20 full-term) at six and 12 months of age was evaluated using the Bayley-III scale. Correlations between cognitive outcome and associated factors were assessed using Spearman correlation. Stepwise multiple linear regression analysis with covariance was applied to identify changes on cognitive score between six and 12 months.

**Results:**

Bayley-III cognitive score in preterm group was significantly lower than in full-term group at both six and 12 months of age. Birth weight correlated with cognitive performance at six months and head circumference at birth at 12 months, in full-terms infants. The occurrence of necrotizing enterocolitis was inversely associated with cognitive score in preterms at 12 months. An increase in cognitive score was observed between six and 12 months in both groups, but the gain was more pronounced in preterms.

**Conclusions:**

These findings suggest some cognitive recovery capacity in the first year despite the restrictions imposed by premature birth and emphasize the importance of early interventions in this population.

## RESUMO

**Objetivo:**

Avaliar o desenvolvimento cognitivo de crianças pré-termo aos seis e 12 meses de idade corrigida e as associações com fatores perinatais e socioeconômicos.

**Métodos:**

O desenvolvimento cognitivo de 40 crianças (20 pré-termo e 20 a termo) foi avaliado aos seis e 12 meses de idade, utilizando a escala Bayley-III. Correlações entre resultados cognitivos e fatores associados foram avaliadas pelo teste de correlação de Spearman. A análise de regressão linear múltipla *stepwise* com covariância foi aplicada para identificar mudanças na pontuação cognitiva entre seis e 12 meses.

**Resultados:**

O escore cognitivo no grupo pré-termo foi significativamente menor que no grupo a termo aos seis e 12 meses. O peso ao nascer foi diretamente associado com o desempenho cognitivo aos seis meses e perímetro cefálico ao nascimento aos 12 meses, nas crianças a termo. A ocorrência de enterocolite necrosante foi inversamente associada ao desempenho cognitivo em pré-termos, aos 12 meses. Verificou-se aumento na pontuação cognitiva entre seis e 12 meses nos dois grupos, porém mais pronunciado no pré-termo.

**Conclusões:**

O estudo sugere que crianças pré-termo apresentam alguma capacidade de recuperação cognitiva no primeiro ano, apesar das restrições impostas pelo nascimento prematuro, e enfatizam a importância de acompanhamento dessa população desde os primeiros meses de vida.

## INTRODUCTION

Preterm-born children are a high-risk population for neurodevelopment delay and cognitive impairment, of which cognitive deficiencies are more frequent.^1,2^ Developmental delays and differences in preterm, compared with full-term infants, are clearly seen within the first year of life.^3^

This setting becomes even more relevant if we consider that the number of preterm births and the survival rates for preterm infants have increased, however, without a reduction in the prevalence of morbidities, including neuropsychomotor alterations after hospital discharge.^3,4^

It is recognized that development of cognitive skills is influenced by biological risk factors associated with prematurity, as well as by socioeconomic and environmental factors.^5^ This highlights the importance of investigating the cognitive development of preterm children in different socioeconomic contexts and environments.

Although previous studies reinforce the interdependent and cumulative action of biological, socioeconomic, and environmental risk factors for unfavorable cognitive outcomes,^5,6^ knowledge concerning the cognitive development of preterm infants within the first year of life and living in developing countries, as well as associated factors, is still insufficient. This context becomes even more relevant if we consider that preterm birth rates have increased worldwide and that most preterm births occur in underdeveloped countries. According to this scenario, Brazil is among the ten countries in the world with the highest number of preterm births, with about 320,000 per year,^7^ which makes Brazilian preterm children a population that should be investigated. Knowing the trajectory of early cognitive development and its associated factors in these children is important to devise better strategies for identifying changes and early intervention.

Therefore, the present study aimed to evaluate cognitive development of premature infants in two moments — at six and 12 months of corrected age — and to examine perinatal and socioeconomic factors associated with the best cognitive trajectories during the first year of life.

## METHOD

This was a longitudinal study involving a cohort of infants born between September 2013 and August 2015. The cognitive development of preterm and full-term infants was assessed using the Bayley-III cognitive scale at the ages of six and 12 months. For preterm infants, we considered their corrected age (CA). The study was in accordance with the Helsinki declaration. All parents signed the informed consent form and the study was approved by the Human Ethics Committee of the Federal University of Minas Gerais (UFMG), Brazil, under the protocol number 369.883 and Certificate of Presentation of Ethical Appreciation (*Certificado de Apresentação de Apreciação Ética* — CAAE: number 12213813.8.0000.5149).

Forty infants participated in this study, allocated into two groups: preterm and full-term. The children from both groups were born between 2013 and 2015 in the maternity unit at the Hospital das Clínicas of the Federal University of Minas Gerais (HC-UFMG). All participants resided in the metropolitan capital region of Belo Horizonte, Minas Gerais, Brazil. They came from low-income families and received free, universal healthcare services by the national public healthcare service. The preterm group included 20 infants recruited from the institution's program for high-risk children called ACRIAR. The ACRIAR is a follow-up service that assists infants born in the maternity unit of HC-UFMG with less than 34 weeks of gestational age or less than 1,500 g. At this service, children receive care from a multidisciplinary team comprised of pediatric doctors, nurses, physiotherapists, occupational therapists, speech therapists, and neurologists. The follow-up is carried out from hospital discharge up to seven years of age according to the following schedule: 1, 2, 4, 6, 8, 12, 18, and 24 months of age, and then annually until the age of seven. Preterm children were recruited from ACRIAR after hospital discharge. The medical records of all children admitted to the referred follow-up service during the study period were analyzed to comply with inclusion and exclusion criteria. Children who met the inclusion criteria were personally invited by one of the researchers who spoke with their parents or legal guardians about the first days of attendance during the follow-up service. Infants with severe sensory impairments (blindness, deafness), cerebral palsy, genetic syndromes, congenital abnormalities, congenital infections, symptomatic congenital heart defects, an Apgar score less than 7 at 5 minutes, muscle tone alterations, intraventricular hemorrhage (IVH) grades III and IV, and periventricular leukomalacia (PVL) were excluded. In order to define these criteria, in addition to the clinical information present in the medical records, the results of the following diagnostic tests conducted in the neonatal period were used: I) For hearing impairment, the result of the Neonatal Hearing Screening performed through the Evoked Otoacoustic Emissions Test (OAE) in neonates at low risk for hearing impairment, or the Brain Evoked Response Audiometry test (BERA) in cases of neonates who were considered at high risk for hearing impairment; II) For visual impairment, the result of the fundoscopic exam performed by an ophthalmologist trained in premature infants’ eye mapping examination; III) For IVH and PVL, the results of transfontanellar ultrasound performed at the corrected age of 40 weeks and conducted according to routine service; IV) For cerebral palsy and changes in muscle tone, results from the clinical records of pediatric neurology visits.

The full-term group included 20 infants born with more than 37 weeks of gestational age and without any known medical diagnosis or atypical development. These children were born in the same maternity hospital and during the same period than the preterm group. The parents were invited in person by one of the researchers during the hospitalization in the maternity ward. Families who consented to participate were asked to provide a telephone contact in addition to signing the consent form, so that the six- and 12-month evaluations could be scheduled. All infants in the control group also underwent transfontanellar ultrasound to exclude cases of brain morphological abnormalities. This examination was performed by the same pediatric neurologist before the children were discharged from the maternity ward.

Cognitive development was assessed using the Bayley-III scale in free-distraction environment. This scale consists of 91 items that assess the sensorimotor development, exploitation and manipulation, relationship between objects, concept formation, and memory. For analysis purposes, the raw scores were converted into composite scores. The Bayley-III was shown to be a valid tool both in research and in clinical practice with satisfactory reliability and validity.^8^ The Bayley-III test-retest reliability range from 0.67 to 0.94, internal consistency coefficients (using the split half method) of 0.87 to 0.93, with moderate to high correlations and measures of similar domains. Each composite score had a mean of 100 points and a standard deviation (SD) of 15 points.^8^ All evaluations were performed by the same researcher, who was previously trained. The assessment was done during a consultation scheduled for this purpose.

The definition of perinatal clinical data and socioeconomic characteristics included in this study was based on the results of a research about factors associated with the cognitive development until the age of five. Socioeconomic data included socioeconomic level and maternal paid employment. Perinatal factors included maternal age, cesarean section, antenatal corticosteroids, gestational age, birth weight, head circumference at birth, small for gestational age, male gender, Apgar score at 5 minutes, days in the neonatal unit, sepsis, retinopathy of prematurity (ROP), intraventricular hemorrhage (IVH) grade I or II, hyaline membrane disease (HMD), bronchopulmonary dysplasia (BPD), invasive mechanical ventilation time (IMVT) and necrotizing enterocolitis (NEC).

Perinatal clinical data were obtained from hospital records and entered on recorded forms. The socioeconomic data were obtained through interviews with the parents and through the Brazilian Criterion of Economic Classification (CCEB).^9^ Briefly, the CCEB is an economic segmentation tool that uses household characteristics (presence and quantity of some household items of comfort and education degree of the household head) to classify population according to average monthly income (in US$) into six groups: A ($3,523 or higher), B1 ($1,917), B2 ($995), C1 ($595), C2 ($407) and D/E (until $285).

The sample size was estimated from a pilot study with 14 full-term and 14 preterm infants, with the same characteristics as the study participants. The statistical power was calculated using the software G*Power 3.1.9.2 to compare independent sample means. The total sample requirement was estimated at 40 infants (20 in each group). In order to reach this number, we assumed an alpha error of 0.05, a power study of 95%, and an effect size of 1.24 (using Cohen's d test) to detect differences in average cognitive score between groups.

We analyzed the results using IBM Statistical Package for Social Sciences (SPSS) 17.0, and the significance level was established at p<0.05. The descriptive statistical variables were presented as means±SD or percentages. The numerical variables were compared through the Mann-Whitney U test for non-parametric data and Student's *t*-test for independent samples for parametric data. Categorical variables were compared by the Pearson's chi-square test. The Bayley-III cognitive score at six and 12 months was compared using independent samples *t*-test.

To analyze the change in cognitive scores from six to 12 months, we used the *t*-test for repeated samples. The correlations between cognitive score and socioeconomic and perinatal characteristics were established from Spearman correlation. In trying to understand the predictors to this evolution in cognitive scores observed in infants at six and 12 months, we applied stepwise multiple linear regression. Cognitive scores at 12 months were set as the dependent variables, while the cognitive score at six months were set as independent variables (sociodemographic and clinical variables, if significantly correlated).

## RESULTS

At six months of age, a cognitive assessment of 54 infants (25 preterm and 29 term) was performed. Among these, 40 (20 preterm and 20 term) returned for evaluation at 12 months. No differences were observed between infants who remained in the study and those who missed follow-up in terms of gestational age (p=0.593), weight at birth (p=0.521), sex (p=0.357); socioeconomic class (p=0.833), and Bayley-III cognitive score (p=0.334).

Descriptive statistics results are demonstrated in Table 1 and include infants who participated in all assessments at both ages. According to cognitive development results, the full-term group presented a significantly higher Bayley-III cognitive score than the preterm group at six and 12 months of age (p=0.002 and p=0.024, respectively).

**Table 1:** Comparison of perinatal, socioeconomic and cognitive development characteristics of preterm and full-term infants.

Measures			Full term group	Preterm group	Comparisons	
					Test	p-value
Perinatal characteristics*						
Birth weight (g)			3213.0±290.6	1603.6±517.9	-11.53†	<0.001
Gestational age (weeks)			39.4±3.5	31.2±1.9	-9.03†	<0.001
Head circumference at birth (cm)			33.7±1.1	28.8±2.4	-8.23‡	<0.001
Male gender			14 (70)	9 (45.0)	-1.60§	0.110
Cesarean delivery			7 (35)	14 (70)	2.76§	0.005
Apgar (5 minutes)		7	0 (0)	1 (5)	1.38	0.711
		8	1 (5)	1 (5)		
		9	12 (60)	13 (65)		
		10	7 (35)	5 (25)		
Small for gestational age			0 (0)	1 (5)	-	-
Antenatal corticosteroids//		None	20 (100)	4 (20)	-	-
		Complete	0 (0)	11 (55)	-	-
		Incomplete	0 (0)	5 (25)	-	-
Sepsis			0 (0)	3 (15)	-	-
Intraventricular hemorrhage grade I or II			0 (0)	5 (25)	-	-
Membrane hialyne disease			0 (0)	6 (30)	-	-
Bronchopulmonary dysplasia			0 (0)	4 (20)	-	-
Necrotizing enterocolitis			0 (0)	4 (20)	-	-
**Socioeconomic characteristics**						
Socioeconomic level (CCEB)¶		B1	1 (5)	2(10)	2.74§	0.098
		B2	9 (45)	4 (20)		
		C1	5 (25)	5 (25)		
		C2	4 (20)	7 (35)		
		D	1 (5)	2 (10)	-0.50‡	
Maternal age			28.2±5.4	27.2±7.1		0.620
Maternal education (years)		< 4	0(0)	2(10)	2.86§	0.582
		4–7	3(15)	2(10)		
		8–11	4(20)	6(30)		
		12–14	10(50)	6(30)		
		≥15	3(15)	4(20)		
Paid maternal employment			13(65)	9(45)	2.37§	0.124
Cognitive development Comparisons (Bayley-III)						
	Cognitive score at 6 mo		115.7±14.2	103.0±10.8	-3.20‡	0.002
	Age(days) of 6 mo assessment		204.2±10.1	193.2±13.2	-2.96‡	0.005
	Cognitive score at 12 mo		120.0±12.0	110.85±12.5	-2.56‡	0.024
	Age(days) of 12 mo assessment		372.6±10.7	376.8±14.7	1.04‡	0.300

* Data are presented in Mean±standard deviation or number (%);

† Mann-Whitney test;

‡ T-test for independent samples;

§ Chi-square test for independent samples;

// A complete cycle of antenatal corticosteroids was considered as two doses of 12 mg intramuscular (IM) betamethasone 24 hours apart or four doses of 6 mg IM dexamethasone 12 hours apart in pregnancies at risk of preterm delivery between 24 and 34 weeks of gestational age, according to national and international protocols;

¶ The CCEB (Criterion of Economic Classification Brazil) stratify the population according to average monthly income into groups: A ($3,523 or higher), B1 ($1,917), B2 ($995), C1 ($595), C2 ($407) and D/E (until $285).

In addition to the characteristics described in Table 1, the children were also evaluated for clinical problems and hospitalizations in their first year. In the preterm group, there were four hospitalizations, with an average duration of 14.7 days (ranging from eight to 19 days), all due to bronchiolitis. In the term group, there were four hospitalizations, with an average duration of nine days (ranging from two to 18 days), for the following reasons: bronchiolitis (2), laryngitis (1), and skin infection (1).

Changes in cognitive scores from six to 12 months are presented in Table 2. We found that, for the total sample, infants showed a statistically significant increase in cognitive score between six and 12 months, and this gain is, on average, 6.1 points (p=0.008). Analyzing groups separately, we observed that the preterm group showed an even greater increase in cognitive scores, with a gain of 7.85 points (p=0.019). The control group had 4.85 points on cognitive score, despite of this gain, it was not considered statistically significant (0.190).

**Table 2:** Evolution of cognitive development from 6 to 12 months in preterm and full-term infants.

	Mean*	SD	SE	95%CI	t	DF	p-value
Full-term group	4.2	13.9	3.1	2.29–10.79	1.36	19	0.190
Preterm group	7.8	13.6	3.1	1.45–14.25	2.57	19	0.019
Total sample	6.1	13.8	2.2	1.64–10.46	2.78	39	0.008

* Mean refers to the variation in the cognitive score between six and 12 months in each group. SD: standard deviation; SE: standard error; CI: confidence interval; t: T test for repeated measures; DF: degrees of freedom.

The association between cognitive development and socioeconomic and perinatal characteristics were also tested. The results are shown in Table 3.

**Table 3:** Correlation between cognitive development and socioeconomic and perinatal characteristics by group.

	Full-term		Preterm	
	6 months	12 months	6 months	12 months
Birth weight (g)	0.18	0.46*	-0.11	0.03
Socioeconomic level (CCEB)†	-0.23	0.08	0.01	-0.19
Maternal age	0.13	-0.43	0.13	0.07
Paid maternal employment	0.24	0.43	0.01	-0.01
Cesarean section	-0.04	-0.23	-0.02	-0.29
Apgar at 5 minutes	0.31	0.17	-0.15	-0.15
Gestational age (weeks)	0.19	0.14	-0.19	-0.20
Head circumference at birth (cm)	0.54*	0.26	-0.29	0.11
Corticosteroids antenatal	-	-	-0.15	-0.02
Days in the neonatal unit	-	-	-0.11	-0.19
Male sex	-0.08	-0.19	-0.21	0.06
Small for gestational age	-	-	-0.06	0.26
Sepsis	-	-	-0.12	-0.08
Retinopathy of prematurity	-	-	-0.05	0.20
Intraventricular hemorrhage grade I or II	-	-	0.06	0.29
Hyaline membrane disease	-	-	0.12	-0.16
Bronchopulmonary dysplasia	-	-	0.06	-0.23
Invasive mechanical ventilation time	-	-	-0.21	-0.18
Necrotizing enterocolitis	-	-	-0.41	-0.50*

* Significant according to Spearman's correlation;

† CCEB (Criterion of Economic Classification Brazil).

Table 4 presents the results of the multiple linear regression analysis. Based on the correlation analysis, we added birth weight, presence of necrotizing enterocolitis and head circumference at birth as independent variables along the cognitive development at six months of age. We observed a statistically significant model (p=0.002) where only the cognitive score at six months of age was a significant predictor of the cognitive score at 12 months of age, explaining 23% of model variance. For each unit added to the cognitive score at six months, there was an increase in the cognitive score of 0.445 units at 12 months of age.

**Table 4:** Stepwise multiple linear regression analysis for cognitive development covariate at 12 months for cognitive development at six months.

	B	SE	p-value
Constant	66.72	14.51	
Cognitive development at six months (Bayley-III)	0.44	0.13	0.002
	R^2^=0.23		

B: Beta; SE: Standard error; R^2^: Explained variance

## DISCUSSION

This study analyzed cognitive performance and associated factors in preterm and full-term infants at an early stage of neurodevelopment. Considering that this period is marked by the emergence of several cognitive and emotional competences,^10,11^ it is important to improve our understanding of the cognitive development and the risk factors associated with prematurity in this period. Furthermore, previous studies showed that preterm infants exhibited poorer cognitive performance compared to full-terms.^12,13^

In this study, preterm group infants demonstrated a lower cognitive score compared to the full-term group at six and 12 months. However, the cognitive scores of preterm infants were within the normal range of the Bayley-III scale, even with a lower average compared to their controls. These results must be analyzed taking into account that the participants included in this study were infants that had a reduced biological risk, considering their mean gestational age and birth weight and that the several risk factors for delays in cognitive development were excluded. Therefore, we emphasize that, although this group of infants had a relatively low, biological vulnerability, differences in their cognitive scores were observed at a very early age. This becomes apparent, especially if we consider that during the first year of life, cognitive development mainly involves skills related to more autonomic functioning, such as sensory learning, perceptual-motor integration, and attention span.^14^ It is only around two years of age (when the period of maturation of attention mechanisms begins and the development of more complex cognitive skills are observed) that cognitive alterations become more frequently identified.^15^

In addition, we evaluated a group of moderately premature infants, whose cognitive outcome is not as explored in research, since most studies investigate the development of infants with a history of extreme prematurity. However, evidence indicates that even these infants, considered to be at lower biological risk, seem to have a higher incidence of school problems, cognitive impairment, behavioral disorders and psychiatric disorders when compared to infants born at term.^16,17^ These deficits appear to remain throughout childhood and could contribute to altered neurodevelopmental trajectories in school age and into adolescence.^13,18,19^ Thus, exploring the cognitive performance of preterm infants in early stages of child development can be useful to support the decision for inclusion in early monitoring and intervention programs.

Considering the clinical factors associated with cognitive development, we observed that head circumference at birth at six months and birth weight at 12 months were directly correlated with the cognitive development of the control group infants. Anthropometric characteristics such as birth weight and head circumference are mentioned in the literature as possible factors associated with neurological development, both in the first year of life^20^ and at school age.^21^

In the preterm group, NEC was inversely associated with cognitive development at 12 months. It should be noted that the history in the neonatal unit and potential complications during hospitalization are associated with adverse cognitive outcomes. Surviving preterm infants with a history of NEC stage II or higher are at risk for long-term neurodevelopmental impairment.^22^

At six months, we observed that the control group was significantly older than the preterm group, with a difference of 11 days, on average. However, as the Bayley-III composite score was pre-adjusted for age and the infants were tested to their maximum level, we determined that this difference did not affect the interpretation of the results.^23^

We also investigated changes in cognitive performance between six and 12 months and found that both groups demonstrated an increase in cognitive scores. However, this increase was statistically significant in the total sample and in the preterm group. This suggests that the increase observed in the cognitive score between six and 12 months of age seems to be a phenomenon more related to the preterm group as a kind of “catch-up” during the first year of life. Cognitive development in the full-term group appears to be more stable during this period. It is important to note that, despite the preterm group showing a more significant gain in cognitive scores between six and 12 months, the average score in the preterm group at 12 months continued to be lower than that of full-term infants at the same age. These results are in agreement with previous studies that reported improvement in cognitive test scores in children with a history of prematurity during early childhood.^24,25^

One possibility for this more pronounced cognitive gain in the preterm group may be a compensatory developmental trajectory.^25^ In preterm infants, this process can be even more pronounced, since the head circumference “catch-up” seems to occur during the first year of life and this process has been associated with better developmental outcomes, as it is directly correlated with increased brain volume.^21^ It is important to consider that the rates of brain growth and development during the first year of life are very high, which cannot be comparable to any other time of postnatal development.^26^ Post-mortem studies and neuroimaging studies have shown that at around 12 months of age, infants reach maximum synaptic density and the pattern of glucose utilization in an infant at this age resembles that of an adult^26^, which coincides with an improvement in children's cognitive and behavioral performance.^27^ In this scenario, the more pronounced gain in cognitive performance observed in the preterm group may suggest the development of alternative compensatory neural networks in response to an injury to the preterm infant's brain, which arise during development and appear to preserve global function through plasticity.^28^ In the same vein, recent long-term studies demonstrate progressive improvement over time in children with no history of IVH grade III or IV or significant white matter lesions, suggesting some capacity for recovery, which still needs to be further investigated.^29^

Another condition that may be associated with this cognitive improvement, observed in the preterm group, was the fact that these infants regularly participated in a multidisciplinary project to monitor their growth and development and referred to the intervention program when necessary. Studies involving children born preterm, and living in urban regions of underdeveloped countries, have shown that cognitive development appears to be substantially benefited by interventions in the early stages of development, such as psychosocial stimulation and pre-preschool experiences.^30^

We also tried to identify predictors for this evolution in the cognitive scores observed between six and 12 months. We found that a cognitive score at six months was the only variable correlated with cognitive performance at 12 months, explaining 23% of the variability in cognitive scores at 12 months. This finding also reinforces the importance of monitoring the cognitive development of preterm children from the early stages of development.

This study has some limitations. First, we studied a small sample size. Therefore, the number of subjects limited the number of analyzed variables associated with the target outcome when using multivariate regression. However, we reached the sample size calculated from a pilot study that included infants with the same characteristics of the studied sample and we obtained both a power study and high effect size. Therefore, we believe that the statistical analysis was not hindered by the small sample size and that the infants included in the present study were representative of the infants generally seen in public health services in our region. In addition, the data collection protocol was extensive, considering the age and attentional span of these children. Thus, the inclusion of a larger number of children than the calculated sample would imply additional costs and the submission of children to a research protocol that could be exhaustive, especially considering their young age. The study sample was, therefore, restricted to infants who suited the inclusion criteria and who attended this service during the study period. Furthermore, we evaluated only two age groups: six and 12 months, which limited our inferences and trajectory designs. Definitively, more research is needed, including more neurodevelopment assessments.

In conclusion, preterm infants demonstrated poorer cognitive performance than full-term infants at six and 12 months old. Anthropometric variables and perinatal complications seemed to be associated with cognitive development at these ages. Between six and 12 months, premature infants presented greater gains in cognitive scores, suggesting some recovery capacity despite the restrictions imposed by premature birth. The results reinforce the need of early assessment of the neurodevelopment and the importance of early intervention.

## Data Availability

The database that originated the article is available with the corresponding author.
